# Whose city is it? Mapping perceived urban livability with citizen-guided AI

**DOI:** 10.1038/s42949-025-00320-x

**Published:** 2026-01-08

**Authors:** Florencio Campomanes, Angela Abascal, Lorraine Trento Oliveira, Monika Kuffer, Anne M. Dijkstra, Alfred Stein, Mariana Belgiu

**Affiliations:** 1 University of Twente, Enschede, Netherlands; 2 Universidad Publica de Navarra, Pamplona, Spain

**Keywords:** Geography, Developing world

## Abstract

Urban livability is shaped by dominant values, often economic or aesthetic, and power dynamics that often overlook the lived experiences of deprived urban area (DUA) residents. As a result, conventional livability indicators risk reinforcing existing inequalities unless these are grounded in inclusive and participatory approaches. To address this issue, we developed lightweight deep learning models – ‘AI-voters’ – trained on livability preferences from both DUA residents and city planners, using open-source satellite imagery. Applied in Ghana’s Greater Accra Metropolitan Area, our approach reduced data requirements to map urban livability by 90% through a two-step urban form sampling strategy that enabled scalable participatory mapping. Training separate ‘AI-voters’ for planners and DUA residents revealed systematic differences: planners not only disagree among themselves but also consistently assign higher livability scores and overlook the preferences of DUA residents, such as avoiding coastal area exposure. The AI-voters mirrored human-voter behavior based on physical urban features such as greenery and building density, especially when trained on the preferences of DUA residents, demonstrating their potential as scalable proxies for local insights. These results highlight the importance of integrating community perspectives into AI models trained to map urban livability to expose hidden spatial inequities and promote more inclusive urban development.

## Introduction

Currently, approximately 1.1 billion people live in deprived urban areas (DUAs)^1^, i.e., areas that are characterized by inadequate access to basic services and infrastructure, poor housing, environmental risks, and social or economic disadvantage relative to other parts of the city^2^. The DUA population is expected to nearly triple in the next 30 years, where Sub-Saharan Africa (SSA) is expected to experience the largest share of this increase with an estimated additional 360 million people living in DUAs by 2030^3^. Most megacities in SSA are predominantly composed of DUAs. For example, in Nigeria, more than 50% of the urban population lives in such areas^4^*-* where overcrowding is extreme. In Nairobi (Kenya), 60% of the population lives in DUAs, occupying only 6% of the city’s land^5^. This intensifies spatial inequalities and places immense pressure on already overburdened urban infrastructures. The DUA population in the Accra Metropolitan in Ghana is estimated to be 50% of the entire urban population^6^, with a large diversity of settlement types^7^.

While several neighborhood upgrading programs have been put in place in some of these cities^8^, the threat of evictions and outright demolitions of DUAs may leave residents entirely without shelter^9^. Such ethically questionable interventions, coupled with alarming demographic projections, suggest that Sustainable Development Goal 11.1, focusing on access to adequate, safe, and affordable housing and to upgrade DUAs by 2030^10^, is increasingly unlikely to be achieved. This lack of progress is further hindered by the persistent exclusion of DUAs from official data and mapping efforts, thereby reinforcing cycles of poverty and marginalization^11^. Such challenges impact the overall quality of urban life^12^ and emphasize the urgent need to reassess how livability is defined, whose needs are prioritized, whose voices are excluded, and how livability assessments can be inclusively implemented.

Urban livability is inherently a multidimensional concept, encompassing diverse factors that influence the well-being and overall quality of life of urban residents^13^. Livability serves as a guiding principle for policymakers^14^ and it extends beyond the provision of basic infrastructure and services. It includes safety, environmental quality, social cohesion, access to cultural amenities, economic opportunities, and a system for participatory governance^15^. Livability is both dynamic and context-specific, with its interpretation varying across cities, communities, and individuals. This subjectivity reflects its inherently cultural and contextual character, challenging the applicability of uniform and top-down planning models^16^. Consequently, urban planning processes should account for the lived experience of diverse communities, including those living in DUAs. Capturing and understanding these diverse perspectives of livability requires access to accurate data and inclusive spatial mapping practices^17^. In the absence of such efforts, communities in DUAs remain invisible, their needs underrepresented, and their voices marginalized. Effective urban development, therefore, requires integrated strategies that combine top-down infrastructure planning with bottom-up, community-driven knowledge to ensure equitable and inclusive outcomes^18^.

**Fig. 1:**
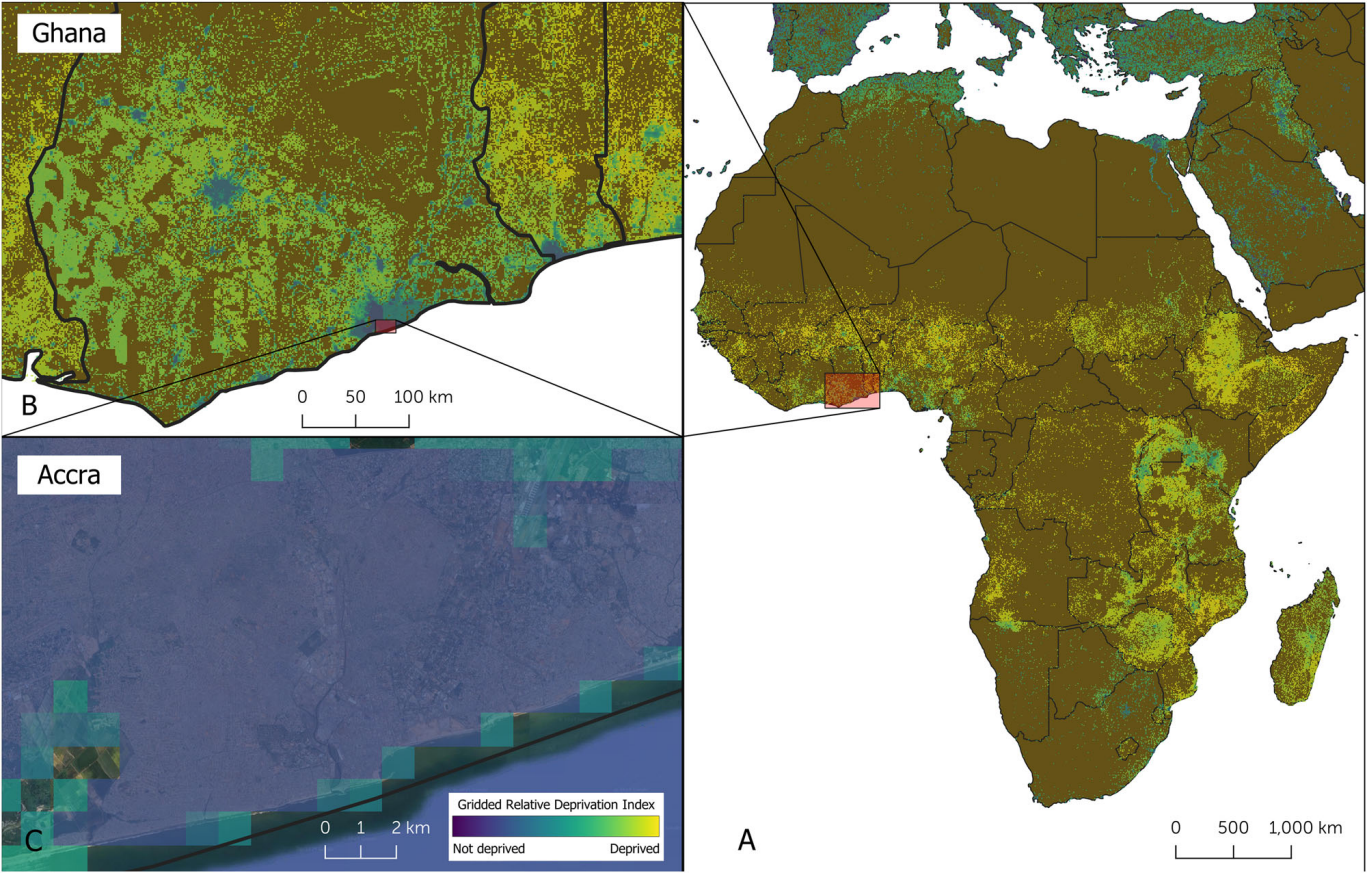
The Global Gridded Relative Deprivation Index (GRDI). combines satellite imagery with sociodemographic data to map spatial patterns of deprivation. It captures broad variations across Africa (**A**) and Ghana (**B**), and provides finer detail within Greater Accra (**C**). In this local context, blue pixels on the GRDI map represent areas with low measured deprivation (GRDI = 0). However, such indicators can be misleading, as they may classify entire neighborhoods as low-deprivation even when deprived urban areas (DUAs) are present within them. This is particularly relevant in Greater Accra, where rich estates and DUAs often exist within the same geographic zones. Greener pixels along the coast and near water bodies indicate higher levels of deprivation. These patterns highlight the limitations of aggregated metrics like GRDI in revealing the complex, localized nature of urban inequality. Data source from the National Aeronautics and Space Administration (NASA) and Columbia University.

Methods for mapping and assessing perceived urban livability (PUL) have evolved in recent years. Early methods predominantly relied on aggregated surveys that provided generalized spatial insights at the district or city level^19^. Such surveys remain valuable for informing urban and regional policy. Yet, they frequently lack the spatial granularity required to capture the nuanced heterogeneity of PUL within urban environments^20^. For instance, Ablekuma Central, situated in the Greater Accra Region of Ghana with a population of more than 150,000^21^, houses both communities with amenities such as swimming pools and highly deprived neighborhoods within close proximity. This intra-urban diversity illustrates the limitations of coarse-scale assessments in accurately reflecting localized livability conditions. For example, the Global Gridded Relative Deprivation Index (GRDI)^22^ shows the majority of the urban area in Accra as not deprived (Fig. 1). To address this limitation, Earth Observation (EO) data have been increasingly used to support analysis at greater spatial detail. When combined with deep learning (DL), EO data can be used to automatically identify key patterns and environmental features that may serve as proxies for the physical dimensions of PUL^23^. Recent studies have demonstrated the utility of DL models to derive information from aerial images, such as demographics, building materials, and green spaces to determine a livability score in a developed country^24^. The trained model was then used to scale the livability assessment across the entire country. The validity and relevance of data-driven methods, however, inherently depend upon the quality and representativeness of their input data^25^. In the context of mapping PUL, this requires data that are representative of the diversity of urban form and that incorporate the perspectives of local populations. Such perspectives may vary significantly and even conflict, as DUA residents may prioritize access to local amenities and safety, whereas municipal planners may emphasize large-scale infrastructure development^26^. Such a divergence emphasizes the importance of methodologies that explicitly accommodate multiple, and at times competing, viewpoints to accurately reflect the complex and subjective nature of urban livability.

A relevant question in the assessment of PUL concerns whose perspectives are represented in the evaluation process. City planners, sometimes together with local academia and non-government organizations (NGOs), are typically the primary actors in local urban planning initiatives and have access to extensive data and technical resources. DUA residents, however, possess equally critical, albeit often underutilized, forms of knowledge. These include localized insights into both the challenges and assets of their communities, derived from lived experience. Moreover, they have experiential knowledge of the entire city as the informal economy makes up about 80% of jobs in African urban centers^27^. Therefore, DUA residents gain significant local knowledge when moving around a city, demonstrating the critical importance of local citizen participation in livability assessments and planning^28^. Moreover, local government planners may have political biases that may not align with the perspectives of DUA residents^29^. Such grounded perspectives are frequently overlooked in externally performed livability assessments^7^.

In recent years, mapping methods that prioritize citizen participation and recognize the subjective and culturally nuanced nature of livability have increased. For instance, pairwise comparisons have been conducted by means of online platforms to expose DUA residents in Nairobi, Kenya, to two very-high resolution (VHR) images of known DUAs and ask them to choose which was a better place to live^24^. These comparisons were used to train DL models and produce a livability score map. Such an approach empowers locals to actively contribute their preferences and priorities for a more livable place, ensuring that their perspectives are central to urban livability mapping. However, this work had several limitations. First, the scope of the researchers’ assessment was limited to only DUAs and did not provide a complete picture of PUL across the entire city. Second, their DL model estimated an exact numerical livability score from VHR imagery, a regression task that is more complex than the binary pairwise selections the livability scores were derived from^24^. Third, the high cost of such VHR imagery poses significant limitations, particularly for applications across broader geographic areas or in resource-constrained settings.

In this study, we develop a multicity-scale framework for assessing PUL by combining satellite imagery, AI, and local contextual knowledge. Using open-source satellite imagery, we develop a novel methodology to quantify and compare differences in livability perceptions between DUA residents and local city planners for large urban agglomerations. The framework consists of three main components: (1) development of a DL-based pipeline for large-scale livability mapping; (2) training of DL models using openly-available satellite imagery and locally collected PUL data of diverse local stakeholder groups and DUA residents; and (3) the identification and analysis of divergences in PUL, to inform more equitable and context-sensitive urban planning practices.

## Results

### Sampling images of distinct urban forms

Pairwise comparison of images (Fig. 3) is a simple and intuitive method to collect local perceptions of urban livability^30^. However, collecting pairwise comparisons for large urban areas, such as the Greater Accra Metropolitan Area (GAMA) in Ghana, is costly and time-consuming. Specifically, an area of 272 sq. km requires 23.1 million pairwise comparisons. To address this challenge, we reduced the number of images for comparison while ensuring that the remaining images represented the broad range of urban forms in the study area. A two-step clustering was employed to select representative image tiles. In the first step, image tiles were grouped according to the dominant local climate zone (LCZ)^31^. In the second step, five urban morphometrics were calculated and their correlation with the DUA reference data was assessed.

**Fig. 2:**
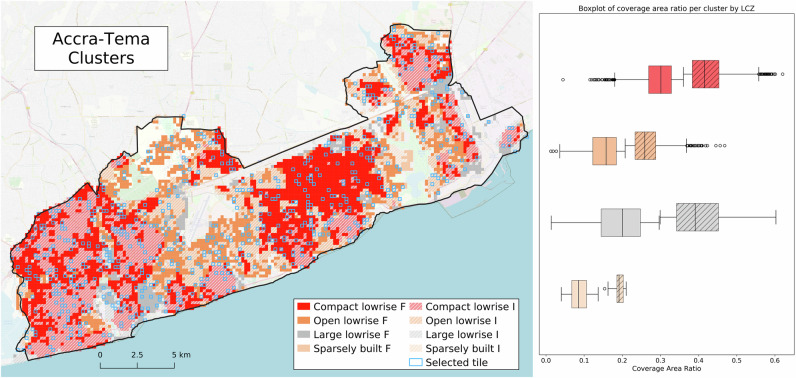
Urban form clusters and selected tiles. The Accra-Tema area was stratified using local climate zones (LCZs) in terms of physical urban form. Each LCZ was then split into two clusters (formal (F) and informal (I)) using the coverage area ratio. The selected tiles are samples representative of the different urban forms and are spread across the entire study area. The value distributions of the coverage area ratio per LCZ and cluster are shown in the boxplot.

Table 1 shows that the coverage area ratio (CAR) and inter-building distance (IBD) had the strongest correlation with the DUAs (r = 0.56 and −0.40, respectively). The CAR was then used as the primary metric for Gaussian Mixture Model (GMM) clustering to discriminate between DUA and non-DUA areas within each LCZ. The GMM clustering showed an overall mean silhouette score of 0.56 (range of 0.40–0.67) for each LCZ, indicating that the clusters were relatively well-defined. Figure 2 shows the spatial distribution of the resulting clusters with the highlighted outlines of the randomly sampled ones (*n* = 660). The accompanying boxplot shows the delineation of formal (*F*) and informal (*I*) clusters per LCZ. We defined these clusters as formal and informal based on their morphological characteristics. The *I* clusters consistently show higher CAR values, indicating that dense urban structures are typically associated with informal areas. These values are especially high for compact low-rise and large low-rise clusters. In contrast, *F* clusters display lower CAR values and represent more formal urban morphologies.

**Table 1:** Urban morphometrics correlations with the total area delineated as DUA per cell

Urban morphometric	Correlation *(p* < 0.001*)*
Coverage area ratio	0.57
Circular compactness	0.24
Longest axis length	−0.39
Rectangular index	−0.35
Inter-building distance	−0.40

### Collecting local contextual knowledge

**Fig. 3:**
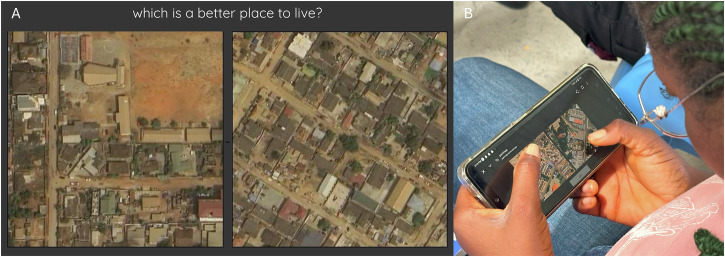
Online pairwise comparison platform in DUA community workshops. Using a web application (**A**), local DUA residents provided their preferences on the better place to live (**B**) and discussed why and how they made such decisions.

**Fig. 4:**
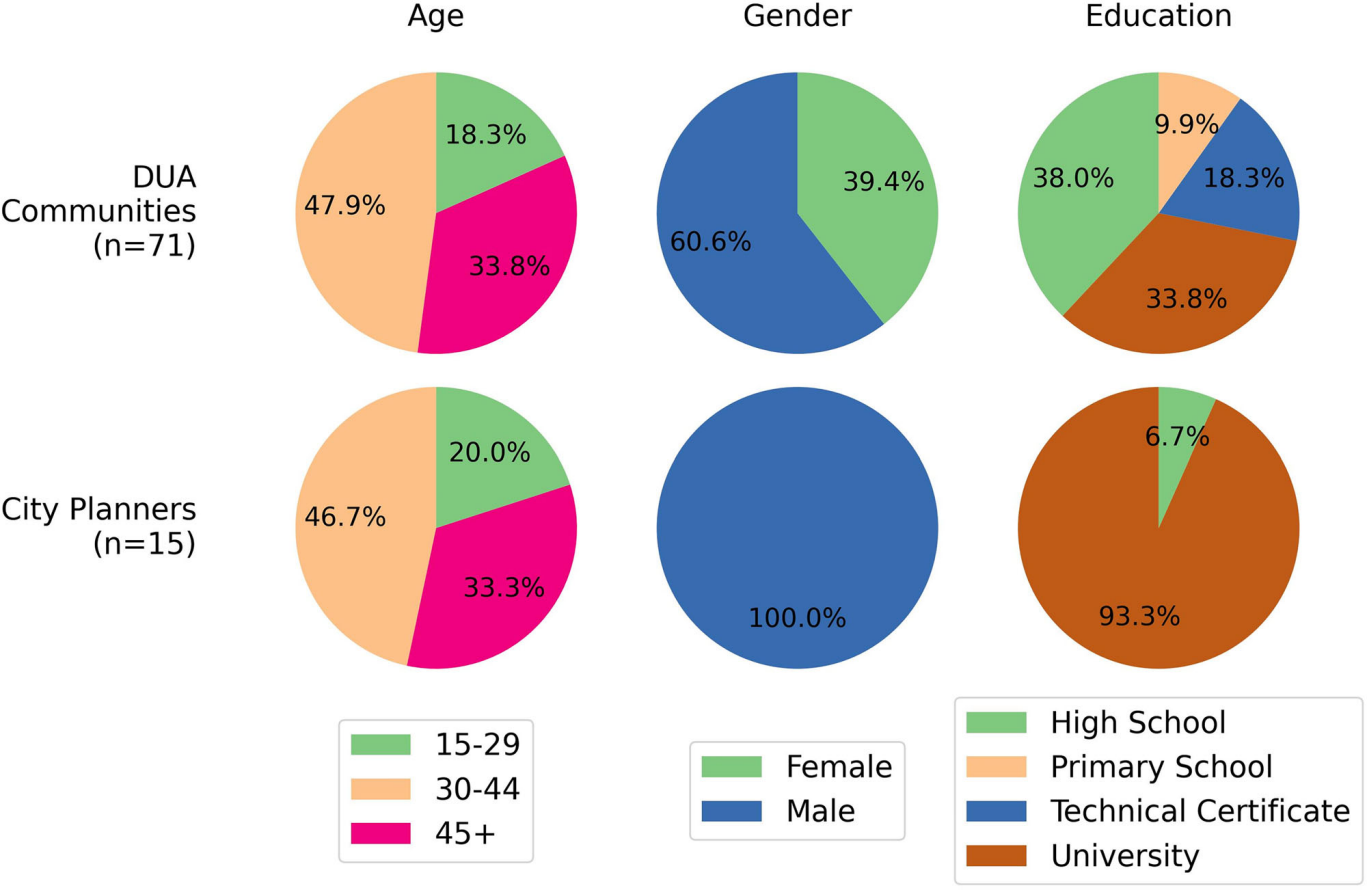
Demographic breakdown of participants across DUA community and city planner workshops.

We collected votes on people’s perception of livability during six workshops in DUA communities (Fig. 3) and one workshop with the city planners of Accra. Three DUA community workshops were conducted in the Accra Metropolitan (Opetekwei, Old Fadama, Accra New Town) and three in the Tema Metropolitan (Zenu, Tulaku, Tema New Town). In total, 71 participants contributed to the DUA community workshops, and 15 participants to the city planners’ workshop. The demographic profile of the workshop participants is seen in Fig. 4.

Each workshop lasted approximately 1.5 hours, thirty minutes to introduce the activity and one hour for the participants to conduct the pairwise comparisons. Overall, we gathered 36,291 unique pairwise comparisons, of which 30,360 came from the DUA communities and 5931 from the city planners.

### AI-voter trained on local perspectives

Three AI-based models, hereafter referred to as AI-voters, were trained with Sentinel-1 (S1) images, each on a different source of votes: one was trained exclusively on the planners’ votes, i.e., pair-wise comparisons, another using the DUA residents’ votes, and the third on a combination of both sources. The AI-voters were trained using a 5-fold cross-validation. Table 2 shows the statistics of the binary accuracy across folds for each AI-voter. The DUA residents AI-voter had the highest mean accuracy of 0.68, whereas the planners AI-voter had the lowest accuracy of 0.62. We observe that the mean accuracy increases as the volume of training data increases. It is important to note that the DUA residents’ AI-voter was trained on five times more pairwise comparisons than the one of planners.

**Table 2:** AI-voter performance metrics per source of training data

Training data	Mean accuracy	SD accuracy	Relative SD accuracy	Number of unique pairs for training
Planners	0.62	0.011	1.80%	5931
DUA residents	0.68	0.004	0.57%	30,360
Combined	0.65	0.010	1.59%	11,862

The mean, standard deviation (SD), and relative SD accuracy of each model are presented together with the corresponding number of unique pairs used in training.

### City-scale livability from different local perspectives

**Fig. 5:**
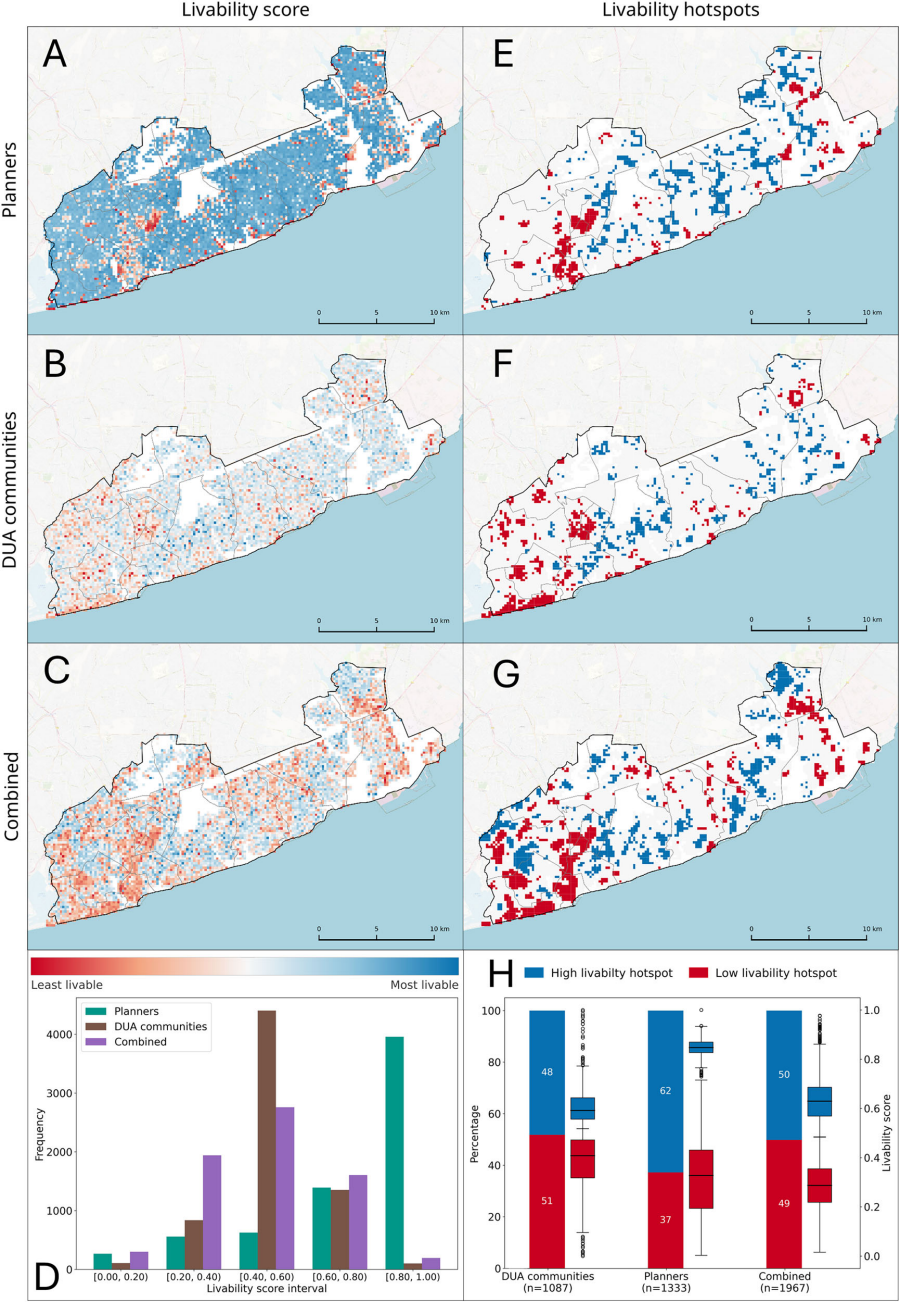
Workshop participants distribution. Demographic breakdown of participants across DUA residents’ and city planners’ workshops. Spatial variation of livability scores (**A**–**C**) and hotspots (**E**–**G**) from three AI-voters. The graphs of the livability scores at 0.2 intervals per AI-voter (**D**) clearly show the similarities and discrepancies between the perspectives on livability of DUA communities, city planners, and a combined perspective. The same is true for the comparison of hot and cold spots (**H**), where planners see more areas as livable compared to the DUA communities. High livability hotspots for planners were found mostly in the central part of the study area, along the transition area between Accra and Tema (Ledzokuku, Krowor, and Tema West), and the northeastern part (north of Ashaiman). For DUA residents, most high livability hotspots surround the Kotoka International Airport, the largest airport in Ghana. Low livability hotspots for planners were in areas with high building density in Accra (Accra New Town and Old Fadama) and Tema (Zenu and Tulaku), while DUA residents considered a large part of the coast with high building density in Accra (Opetekwei) as a low livability area.

Building on the three trained AI-voters, we used each model to identify which of the two areas was the better place to live, given a set of image pairs created from all the tiles across the study area. In total, ~860,000 pairs were created, and for each pair, each AI-voter independently selected which image was the better place to live. The output choices from each AI-voter were subsequently processed by means of the rating algorithm TrueSkill^32^. TrueSkill transforms the choices into numerical ratings, which are then normalized to a scale ranging from 0 to 1 to represent PUL scores (Fig. 5A–C). The PUL scores from the city planners (Fig. 5A) had a mean score equal to 0.72, considerably higher than the DUA residents’ mean PUL score of 0.52. PUL scores from DUA residents (Fig. 5B) were mostly between 0.4 and 0.6 while most PUL scores of the planners were between 0.8 and 1. The PUL from the combined perspective had a lower livability average of 0.48 and displayed a normal distribution (Fig. 5D). This combined view effectively balanced the planners’ more optimistic assessments and the moderate evaluations from DUA residents. Notably, the greater spread of scores beyond the 0.4–0.6 range in the combined dataset enhanced the visibility of spatial disparities in livability across the city (Fig. 5C).

A hotspot analysis revealed similar patterns to those of the livability scores (Fig. 5E–G). Among the 1333 tiles identified as a livability hotspot by planners, 62% were classified as high livability. Conversely, the DUA residents (1087 tiles) and combined (1967 tiles) PUL had a nearly even distribution of significant clusters between high and low livability. The hotspots are relative to the livability values within each specific perspective. Fig. 5H shows that there are substantially higher livability scores in the high livability clusters from the planners (median = 0.85) than those from the DUA residents (median = 0.59) and combined (median = 0.63). These findings indicate that a cluster deemed highly livable by DUA residents may not meet the same threshold from the planners’ standpoint. Likewise, low livability clusters identified by DUA residents were generally associated with higher scores than those recognized by planners. Some of these low livability clusters were in DUAs. Although these areas still had low livability scores relative to the entire city, residents perceived them more favorably than planning professionals did.

**Fig. 6:**
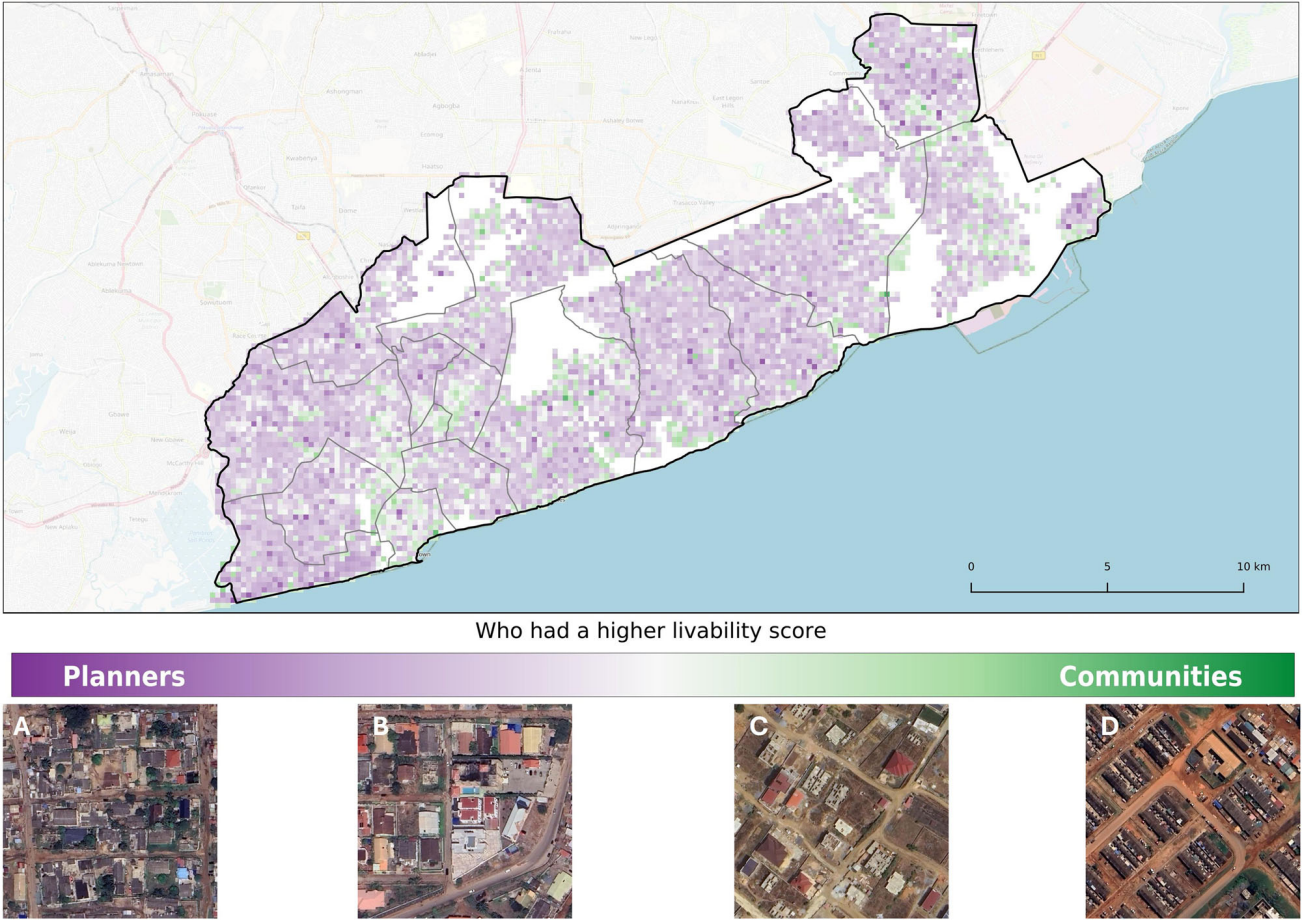
Raw differences of livability perspectives. Planners generally had higher PUL scores compared to DUA residents/communities across the entire study area. Such areas were **A** those with organized (mostly paved) roads and medium-sized buildings that were not too close to each other and **B** large and uniformly laid out buildings near the highways. The areas where communities had higher PUL were **C** sparsely laid out medium-sized buildings and **D** similar-looking buildings in a grid layout.

**Fig. 7:**
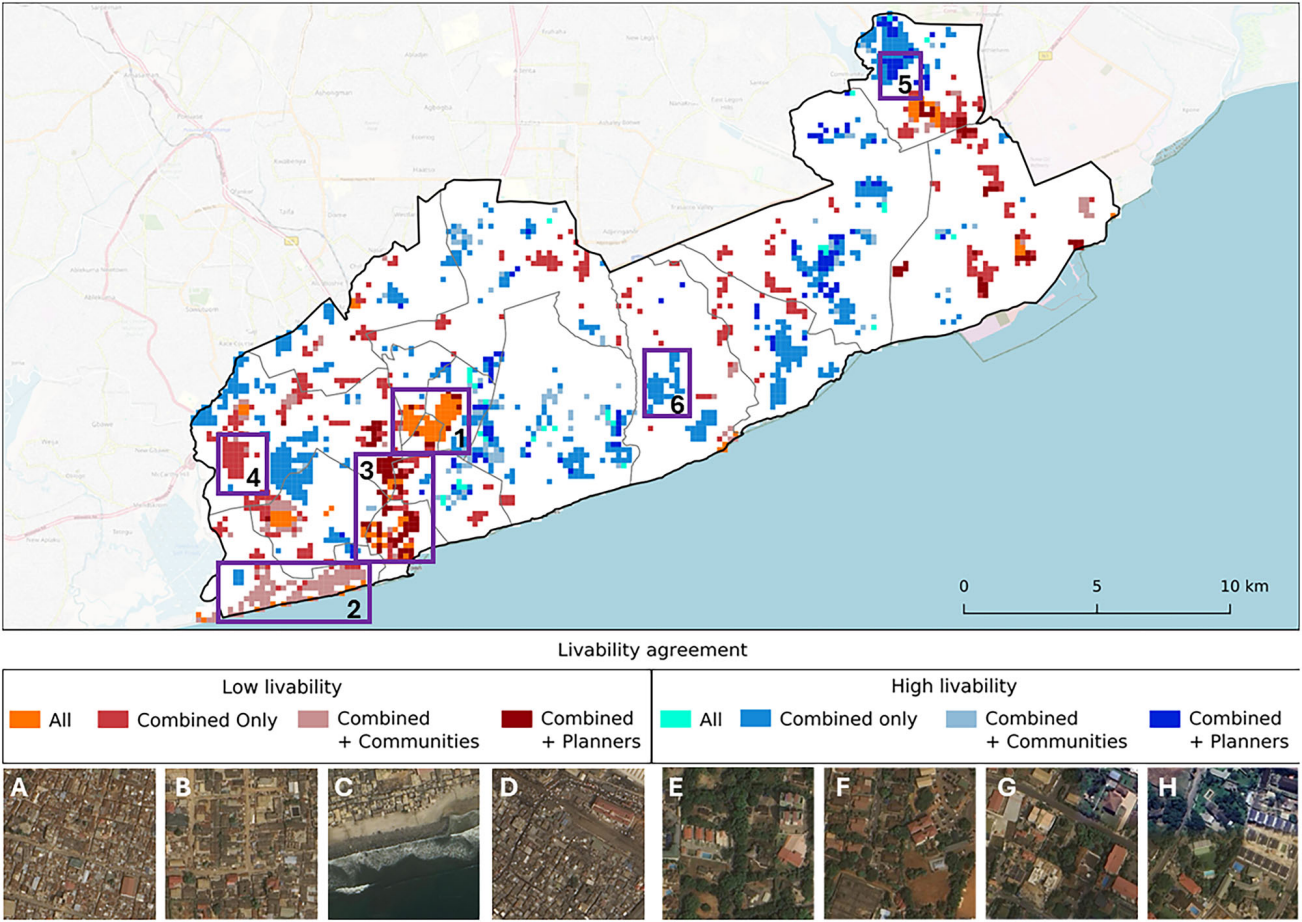
Level of agreement in perceived urban livability clusters. Low livability hotspots across all perspectives (planners, DUA residents, combined) were mostly **A** dense urban areas while their differences were in the surrounding environments. **C** Dense areas near the coast were not preferred by DUA residents, and **D** dense areas along creeks or rivers were not preferred by planners. In the combined perspective, **B**, several low livability hotspots not found in both planners’ and DUA residents’ perspectives emerged, which were also existing known DUAs. For high livability hotspots, the agreement across all three perspectives was minimal, and concentrated in **E** wealthy estates with dense greenery and amenities like swimming pools. Areas considered highly livable by DUA residents/communities **G** focused more on the layout of buildings while planners had preferred **E** more greenery. Many new high livability hotspots emerged from the combined perspective (**F**) and featured medium-sized buildings and a high level of greenery.

We next quantified the differences and similarities between the livability perspectives of planners, DUA residents, and both combined. Planners assigned higher livability scores than DUA residents in 82% of the tiles (*n* = 5600) and, of that, 63% (*n* = 3555) scored higher than DUA residents by 0.25 to 0.5 (Fig. 6). Nonetheless, some areas demonstrated concordance between the two groups. For instance, Accra New Town (Figs. 7–1), a densely populated DUA, was identified as a low livability cluster by both groups. In contrast, divergence was noted in coastal DUAs (Figs. 7–2), which were classified as low livable by residents but not by planners, suggesting that DUA residents may not want to live near the coast. The opposite was observed along the river, where planners identified low livability clusters that DUA residents did not (Figs. 7–3). The combined perspective not only reflected similarities with both DUA residents’ and planners’ perspectives but also revealed new livability hotspots, both low (Figs. 7–4,B) and high (Figs. 7–6,F). Areas identified as high livability clusters, by planners were generally situated between central Accra and Tema. Finally, residential zones adjacent to the main airport, characterized by lower building density and greater green space, were regarded as high-livability areas by both residents and planners.

### AI-voter follows human voting behavior based on physical features

**Fig. 8:**
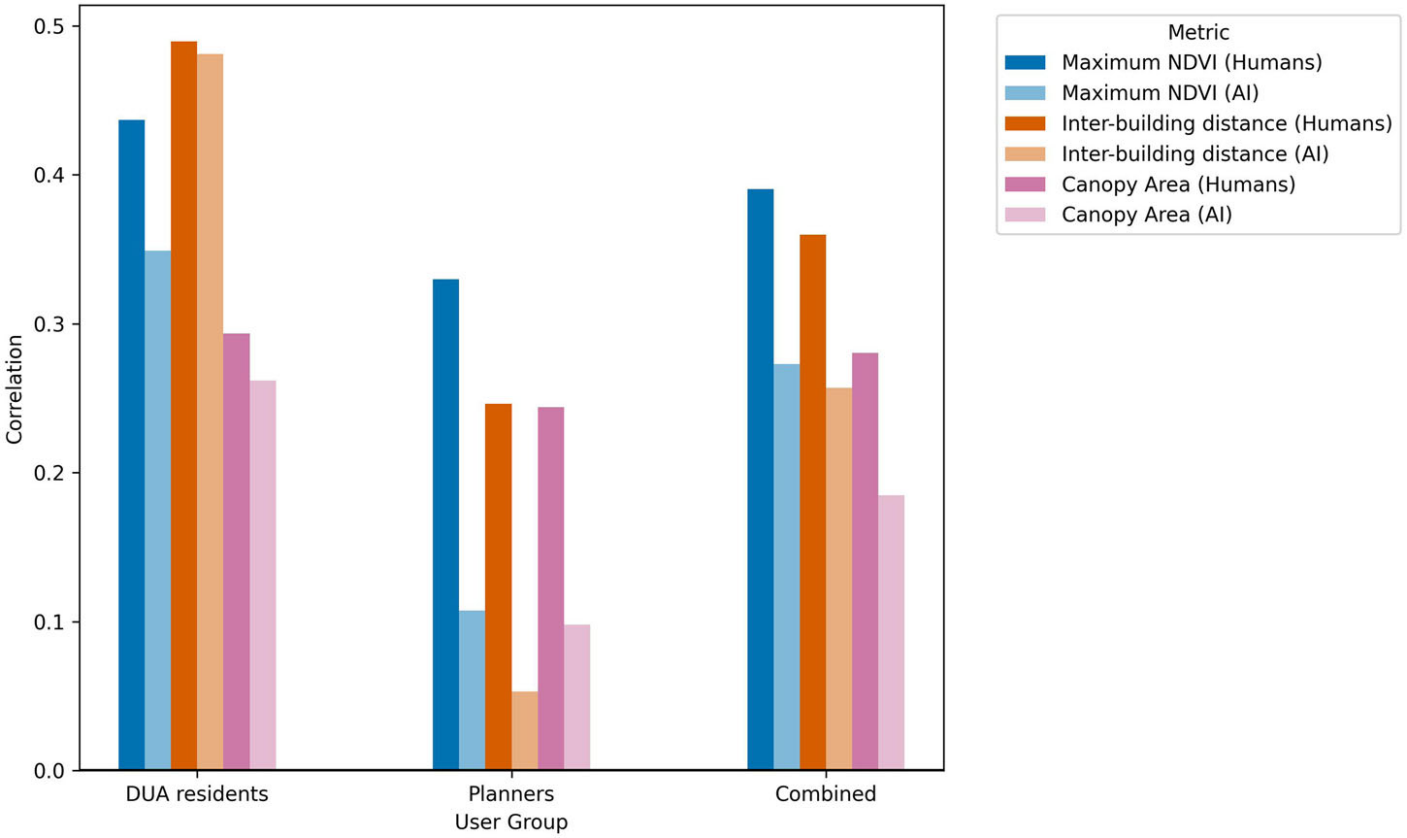
Relating voting behavior of humans and AI to physical features. Correlations between a tile’s winning ratio and three physical features are shown. Darker shades represent correlations with human votes, and lighter shades represent correlations with AI-generated votes. Overall, the AI-voter’s behavior more closely mirrors the correlations observed for DUA residents than for planners.

We compared the voting behaviors of the different perspectives: DUA residents, planners, and both combined, with their AI-voter counterparts. Voting behavior was quantified as a ratio of the number of times an image tile was chosen as a more livable place to the total number of times it appeared in the voting scenarios. This ‘winning ratio’ was then examined in relation to three key physical urban features: maximum NDVI (Normalized Difference Vegetation Index), IBD, and total canopy area (TCA). Our findings indicate that the AI-voters, though not in perfect alignment, mirror the patterns observed in human voter behavior and this was more apparent with the DUA

residents than with the planners (Fig. 8). Maximum NDVI and TCA, both proxies to green space, were positively correlated with voting behavior across all perspectives, for both human and AI participants.

Insights from the participatory workshops revealed that green space was consistently cited as a major factor influencing perceptions of livability for both planners and DUA residents. Maximum NDVI had a higher correlation with voting behavior than TCA likely because NDVI represents a broader representation of green space, whereas TCA captures only tree coverage and may overlook other vegetated open spaces that nonetheless contribute to perceived livability. IBD was also a popular criterion for a better place to live during the workshop discussions. Several participants highlighted the concept of “ventilation” referring to the space between buildings, which facilitates airflow. With higher IBD, the density of buildings in the area decreases causing more space for “ventilation”. Areas with greater IBD are typically less densely built and thus perceived to offer better environmental quality.

We examined the correlations between physical features and voting behavior across different perspectives and voter types. For planners, these correlations were generally weaker than for DUA residents or for the combined perspective. The weaker correlations were particularly evident in the AI-voter simulations compared to human voters. For DUA residents, the AI-voter correlations closely matched those of human voters, reaching 80% to 98% of the human correlations. In contrast, the AI-voter correlations for planners were much lower, ranging only from 21% to 40% of the human correlations.

## Discussion

Our key findings demonstrate that integrating local knowledge, openly available EO data, and data-driven methods – including the novel development of an AI-voter – enables spatially detailed and multicity-wide mapping of PUL. This integration marks a significant step forward in extending participatory urban analysis beyond neighborhood-level case studies to broader, multicity-scale applications. We identified significant discrepancies in PUL between different groups emphasizing the inherently subjective and context-dependent nature of urban livability. Finally, our findings show that urban livability, as perceived by its citizens, can be mapped at scale with free-cost EO data.

Quantifying local perspectives of PUL using pairwise comparisons and EO can easily become a costly and exhausting task when scaling up to city-scale or larger areas for two reasons: the number of images needed to be compared can become prohibitively large, and suitable EO data is not always readily available^30^. Our method addresses both limitations. To ensure the feasibility of large-scale comparison, it was necessary to reduce the number of image tiles while maintaining a sample set representative of urban form. Without such optimization, some image tiles would remain unpaired in the comparison process, resulting in unranked tiles with no corresponding livability score. Our 2-step clustering using LCZs and urban morphometrics allowed for representative sampling of both Accra and Tema, reducing the number of images needed for comparison by 90% while maintaining the images’ diversity of urban forms. We found that the CAR had the highest correlation with the DUAs in the GAMA region, confirming that urban density is indeed an important indicator of deprivation or reduced livability in the area^6^. However, this relationship may not hold in less densely developed regions, where indicators such as street network irregularity or building heterogeneity may be more reflective of informality and deprivation^33^.

The EO data presented to the respondents has its limitations. The VHR satellite imagery does not provide a human-eye level view, unlike street-view (SV) images that are often unavailable in DUAs. This may have an impact on how respondents assess the livability of an area. For instance, the presence of people cannot be explicitly seen in satellite imagery and only inferred from the presence of residential buildings. Hence, our use of EO data primarily captures urban density as a proxy for urban form and general informality, but it inherently misses dwelling-level overcrowding, a key feature of deprivation. Apart from the presence of people, the physical features present in the area, such as greenery and building density, distinguishable from EO data, play an important role in livability assessment.

To scale this method beyond the GAMA region and ensure consistent EO coverage, we relied on openly available S1 radar imagery to train our AI-voters. We used two dual-polarizations, namely vertical-vertical (VV) and vertical-horizontal (VH), that are particularly relevant for urban settings^34^. The use of these two radar polarizations, instead of multispectral (4 band-RGB, near-infrared) satellite imagery, makes image interpretation difficult^35^. The tradeoff is that radar is able to penetrate cloud cover, detect vertical structures better^36^, and is sensitive to surface texture^34^. Given the high cloud cover in tropical regions like Ghana^37^, it is critical to have data that is not hindered by clouds. Even though S1 supports broad spatial coverage, there is a potential limitation associated with its relatively coarse spatial resolution of 10 m that may not be able to capture the fine-grained spatial detail necessary to distinguish which of two urban locations offers a better place to live. Also, this resolution led to input images of 20 × 20 pixels for the AI-voter, thus constraining the range and complexity of neural network architectures employed in this study. High-performance models such as vision transformers^38^, however, require larger images to fully leverage their representational capabilities.

Our AI-voter performed well as compared to other studies that had access to more samples and more detailed information. For example, previous work using street-view and models trained on 370,000 pairs for a similar pairwise comparison task, achieved binary accuracies between 0.64 and 0.73^39^. In contrast, our AI-voters were trained on less than one-tenth of the training volume used in such prior research (6,000 and 30,000 image pairs). Despite the smaller sample size, the images used in creating the pairs could capture the diversity of the urban forms present in the study area because of the cluster sampling strategy implemented in our study. This outcome concurs with data-centric artificial intelligence (DCAI) where representativeness of input datasets is more vital than volume in ensuring good model performance^40,41^.

The AI-voter’s capacity to uncover hidden urban preferences was critical for scaling the analysis from a localized neighborhood scale to a broader, multi-city context. Given the impracticality of collecting sufficient pairwise comparisons for all 6,800 candidate images, the AI-voter, trained using 10% of the dataset, was able to simulate pairwise comparisons for the remaining images. In its absence, livability scores would have been available for only a small subset of locations. Moreover, the decision to frame the task as a binary classification rather than as a regression problem proved effective. Unlike previous work^30^, our method yielded comparably robust outcomes despite using less complex models and less detailed image information.

Existing literature has proven that PUL divergences exist between the livability perspectives of residents and city planners^42^. We observed generally more favorable assessments from the planners, the first time this was empirically demonstrated at this scale. Most of the high livability hotspots of the planners were in the transition area between the city centers of Accra and Tema (Fig. 5E), as these areas were less congested. This was not the same for the DUA residents as they preferred to live in the greener areas surrounding the airport, featuring medium-to-large-sized buildings and paved roads (Fig. 5F). The emergent hotspots in the combined perspective (Fig. 5G) likely come from the relative nature of hotspot detection, where balancing perspectives alters the thresholds for identifying hotspots. In the low livability hotspots from the DUA residents’ perspective, some of which matched the locations of known DUAs, the median livability scores were higher than those from the planners’ perspective (Fig. 5H). This discrepancy may reflect DUA residents’ stronger sense of belonging^43^ and familiarity with local job opportunities within DUAs, which were mentioned several times during the workshops. These divergent perspectives emphasize the critical importance of incorporating diverse viewpoints and local experiential knowledge into planning processes, as they have direct implications for the formulation and implementation of equitable urban policies.

Our findings stress the need for planning frameworks that explicitly account for and address the perception gaps across different socio-demographic groups. Pipelines and tools such as the one developed in this study offer valuable potential by enabling the simulation of how proposed urban interventions might be received by distinct stakeholder groups. Such early-stage insights can play a pivotal role in ensuring that urban development initiatives do not exclude or marginalize vulnerable populations.

The resulting maps can be used by planners to better understand the DUA residents’ preferences and identify unmet needs in DUAs in terms of physical features. Our goal is not to select a single best planning perspective, but to offer a shared evidence base for inclusive and informed stakeholder dialogue.

Establishing a shared understanding of discrepancies and convergences in livability perspectives, as demonstrated in Fig. 7, represents a step towards more equitable urban development. The figure further illustrates how the combined perspective serves as a potential middle ground between DUA residents and planners, while simultaneously highlighting the priorities expressed in each individual perspective. We amplify the often-overlooked views of DUA residents by mapping their preferences (i.e., Figs. 7–2) to reveal what they value and what they find lacking about their neighborhoods. Our results offer a starting point for transparent, citizen-driven, and evidence-based discussions between local stakeholders, including both city planners and DUA residents.

The voting behavior of the DUA residents, planners, and altogether, and their corresponding AI-voters showed similar but imperfect alignment in relation to physical features. Tiles with higher natural greenness (NDVI and TCA) were moderately more likely (correlation 0.3–0.5) to be selected as better places to live, reflecting DUA residents’ workshop feedback. This coincides with localized findings that the presence of urban nature, or greenery in urban areas, leads to more physical activity and better health outcomes^44^. Previous findings in Paraguay otherwise suggest that urban greenery in informal areas corresponded to lower life satisfaction as DUA residents may have connected urban greenness to unmanaged waste dumps or hazard-prone areas near rivers or steep slopes^45^, emphasizing the context-dependency of livability. Our findings related to urban density, in the form of IBD, and its positive correlation to livability in Accra and Tema, is in agreement with previous work that people from Sweden who lived in the most dense and highly urbanized areas were at higher risk of developing depression^46^, suggesting that urban density may be more generalizable across spatial contexts than urban nature. Given the decline in urban greenery exposure in the Global South^47^, interventions aimed at improving livability using urban nature should consider that its mere addition and presence is insufficient if it is of low quality or left unmanaged.

When assessing how well the AI-voter captured its human counterpart’s voting behavior, we found that correlations with physical features were consistently lower than those of human voters. This was anticipated, as the AI-voter was not intended to perfectly simulate human voting behavior. However, these differences varied largely across user groups. For instance, the planners’ AI-voter’s correlations to physical features were only 21 to 40% of their human counterparts, whereas the DUA residents’ AI-voter correlations to physical features closely matched its human counterparts, reaching 79% to 98%. These findings suggest greater variability in planners’ assessments, potentially reflecting divergent professional judgments or unmeasured criteria. Many planners drew from their diverse backgrounds to interpret multiple layers and meanings in the images beyond density or open space, particularly when they could mentally locate the tile within the city. This high variability likely limited the planners’ AI-voter in capturing consistent relationships with physical features.

Although a larger sample of city planners could strengthen the analysis, the subjective nature of PUL implies that additional votes would not necessarily increase consistency in their assessments. Nevertheless, the relatively high accuracy of the planners’ AI-voter (Table 2) indicates that it can make reliable decisions, even if these decisions depend on features beyond those included in our analysis.

Our study treats each participant group as internally homogenous, despite the possibility of intra-group variation driven by cultural, contextual, and individual-level factors. Livability, as a construct, is inherently subjective and context-dependent, and may therefore differ widely even within seemingly uniform groups^48^. In areas like the GAMA region, factors such as historical significance, community networks^49^, and informal economic activities^27^ deeply influence how residents perceive their neighborhoods. For example, DUA residents’ views of coastal areas as low livability zones could be tied to environmental risks or lack of social infrastructure, factors that might be undervalued by planners. Historically, planners overlook the significance of these contextual factors, such as cultural connections or local knowledge, which lead to planning interventions that exacerbate racial and economic inequalities^50^. The diverse ways in which residents and planners interpret the same areas emphasize the need for planning tools that integrate not only objective metrics but also the rich, lived experiences of local populations^51^, i.e., subjective community insights, providing epistemic justice in urban planning. Future research could further explore the causes of these PUL discrepancies through more intimate settings like focus group discussions, expert interviews, or walking interviews, enabling more inclusive and contextually grounded urban development practices.

To our knowledge, this study represents one of the first attempts to map and analyze diverse PUL perspectives using AI and freely available satellite imagery. By training models to reflect the PUL of different participant groups, we demonstrate how AI can serve not only as a technical tool but also as an analytical framework for interpreting subjective urban design preference. In this study, we focused on two stakeholder groups, namely DUA residents and city planners. However, the approach is flexible and could be extended to reflect other socio-demographic perspectives. For example, AI-voters could be trained to capture PUL differences across dimensions such as gender, age, educational level, or a combination. Such stratifications have already been instrumental in revealing how different segments of the population evaluate urban landscapes^52^.

Our work aligns with data-centric AI^53^ and only tangentially with emerging trends in AI for urban remote sensing, where much of the focus lies in multimodal data and model-centric approaches such as foundation models (FMs) and self-supervised learning (SSL)^54^. Despite the fact that FMs and SSL excel in objective tasks (e.g., road or building detection), more complex and subjective tasks such as assessing PUL require tailored data-centric AI models with locally grounded datasets to capture contextual nuances accurately^53^. Though FMs and SSL offer scalability, their reliance on large-scale, often biased training data^55^ risks misrepresenting local subjectivities^56^. Furthermore, we argue that fully automating AI pipelines for subjective applications (e.g., urban perception) faces inherent limitations, as local knowledge, particularly from underrepresented groups like DUA residents, remains indispensable. In some African cities, DUAs account for up to 90% of the urban population, making their inclusion critical for any meaningful urban analysis. Moving forward, we urge prioritization of the collection of high-quality perception data (e.g., PUL) through citizen science in marginalized regions, like DUAs, across diverse global contexts. Such data will enable testing of the AI-voter’s transferability that can increase the spatiotemporal scalability of PUL mappin,g resulting in more inclusive and equitable urban planning globally.

Ultimately, tools like AI-voters are not intended to replace participatory planning processes altogether but rather to enhance them. These tools offer a scalable, data-driven means of quantifying and visualizing diverse perspectives, complementing more traditional community engagement, which is very localized and covers only a few areas. Rather than relying exclusively on subjective assessments or limited participation, AI-voters can scale up local knowledge across larger urban areas and amplify marginalized voices in the urban planning process. By integrating AI-based insights with qualitative discussions, planners can more effectively identify areas of consensus and divergence, thereby fostering more inclusive and equitable urban development. In this way, the findings of this study lay the groundwork for a more participatory and data-driven approach to urban planning that considers the nuanced experiences and preferences of diverse urban populations.

## Methods

### Data and study area

In the Greater Accra Region in Ghana, the legacy of colonial and discriminatory urban planning, military installations, migrant settlement patterns, and rapid urbanization has resulted in spatially segregated and unequal neighborhoods^57^. Many existing studies have mapped urban poverty in Accra, the primary urban area and capital of Ghana^6,7^. Yet only a few have focused on secondary cities like Ashaiman and Tema, which have seen a surge in growth more recently^6^.

Our study area consisted of the urban residential areas in the Greater Accra Region, from the western part of the region in the city of Accra to Ashaiman and Tema in the eastern part, which covers 272 square kilometers. We divided the area into 200-meter by 200-meter tiles, resulting in 6800 tiles. Previous studies have used 100-meter tiles for livability or deprivation mapping^30,58^, but to ensure the computational feasibility of our method while adding more contextual information, we opted for 200-meter tiles. A larger tile would run into areas of more heterogeneity within a single tile. Our choice of tile size also has a limitation that broader features, like an area’s proximity to large-scale infrastructure networks, such as major roads and highways, may not be visible. Also, a larger tile does not pinpoint specific households and avoids potential misuse of results. These tiles served as the main extent of all spatial datasets and imagery.

In this study, we used S1 radar images from 2022 clipped to the 200-meter tiles. Another reason for the 200-meter tile size was to allow for a larger image, given the coarse spatial resolution of S1 (10 meters). Since our aim was to scale this approach across large areas, we used S1 images as these are openly available, cover the entire globe, have a 5-day revisit period, and are weather-independent^59^. S1 is useful for detecting objects present in urban settings like settlements, bridges, green spaces, and water^34^. These detectable objects are of general importance in determining urban livability, hence the use of S1 images.

Urban form, characterized by the layout of buildings and roads, is a factor that partially determines PUL^60^. In our study, we quantified derived urban morphometrics (UMMs)^61^ calculated from the Google Open Buildings v3 (GOBv3) dataset^62^. Specifically, we calculated several UMMs and specifically focused on coverage area ratio (CAR), circular compactness (CCO), longest axis length (LAL), rectangular index (REI), and inter-building distance (IBD), as they had higher correlations to DUAs. CAR and IBD relate more to building density, while CCO, REI, and LAL mostly describe layout irregularity.

### General workflow

**Fig. 9:**
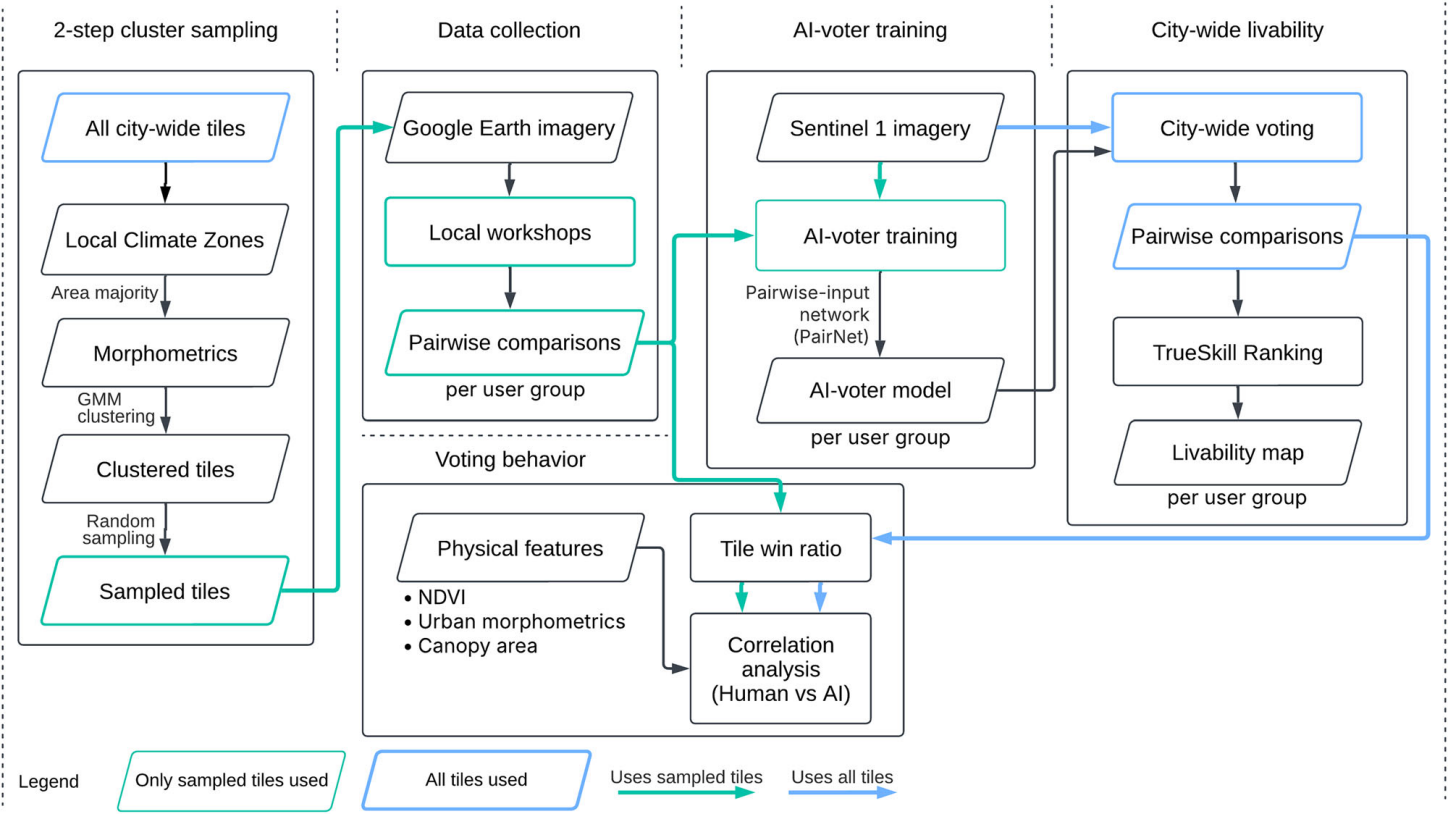
Methodology overview. First, we take a subset of image tiles representative of the urban forms present in the study area to be used in the data collection. We then collect pairwise comparisons from this subset of image tiles through local community workshops. The collected pairwise comparisons are used to train an AI-voter to make pairwise comparisons of other unseen images from the rest of the study area. These are then used to calculate perceived urban livability scores. This is done for each user group (DUA residents, planners, combined). The AI-voter selection behavior is compared to its respective user group behavior vis-à-vis physical features like green space and density.

This study was conducted in four phases, as shown in Fig. 9. First, we determined a practical, feasible, and representative subset of tiles to be chosen for citizen evaluation. To do so, we performed a 2-step cluster sampling method across the study area. This representative sample was then used in the data collection phase to create a set of pairwise comparisons. During local workshops, two user groups – DUA residents and city planners – voted on these pairs. Their votes, combined with the respective S1 imagery, were used to train separate AI-voter models for each group. Finally, we applied the AI-voter models to predict pairwise comparisons across the study area. The predictions served as inputs for TrueSkill, which generated the final PUL map.

### 2-step cluster sampling

The number of image tiles across the study area (6800) was already a large number, given that there would be approximately 23.1 million possible unique pairwise comparisons. This is far too large a number to feasibly collect in the field. For reference, it took about 3 months to collect 1 million pairwise comparisons from DUA residents^30^. Hence, a subset of these image tiles needed to be selected. The subset would be shown to the locals for pairwise comparisons in the field. To ensure that the locals saw a diverse set of image tiles, we sampled a subset that would be representative of the different urban forms within the study area since urban form has been shown to be a relevant factor to livability^60^. We used the local climate zones (LCZ) dataset^31^, a globally available 100 m classification of urban (10) and natural (7) typologies. We focused on the urban land cover (ULC) classes. These ULC classes describe the urban form of the area, like the density and size of buildings. We calculated the area of all ULC classes within each 200 m tile, and the class that covers the most area would be assigned to that tile. The goal was to have sampled tiles including both formal and informal areas. However, upon visual inspection, we realized that within each ULC class, there was still a mix of clearly formal and informal areas, the separation of which was not the original aim of the LCZ dataset.

To further decompose each ULC class into formal and informal, we performed a Gaussian mixture model (GMM) clustering with 2 clusters for each ULC class. GMM can handle clusters of different sizes, shapes, and orientations. Moreover, we assumed that there were only 2 clusters (formal and informal) within each ULC class; hence, we did not need to conduct an assessment for the optimal number of clusters. To select the metric for the basis of the clustering, we conducted a correlation check with an existing reference dataset of DUAs for Accra^7^ and five urban morphometrics^61^ as these showed to be associated with deprived or informal neighborhoods^63^. We calculated the correlation between the total area of DUA per cell and the averages of the urban morphometrics. CAR and IBD showed strong correlations. To avoid potential multicollinearity effects, IBD was excluded due to its comparatively weaker association than CAR. We calculated the average CAR value per cell and subsequently used this in the GMM clustering to be able to separate between formal and informal areas per LCZ. To assess the separability of the resulting clusters, the silhouette score^64^ of each cluster was calculated and averaged. This score represents the closeness or separation of data points in one cluster, where higher values mean the data points of that cluster are closer to each other. From these clusters, we randomly sampled approximately 10% of the tiles (with a minimum of 1 tile) to ensure that each cluster was represented in the pairwise comparisons. Ideally, images from the ground, such as street view (SV) images or 3D model,s would give a more realistic view of an area, but with their limited availability, especially in DUAs, Google Earth (GE) images were used instead. GE images of the randomly sampled tiles were masked, and these would serve as the images to be voted on by the locals. We chose not to use street view images, despite their easier interpretation, as their spatial coverage in deprived areas is limited.

### Collecting pairwise comparisons from locals

We conducted six workshops across DUA communities in Accra, Tema, and Ashaiman, and one workshop with city planners and experts from Accra. In the workshops, we first allotted 15 minutes for the participants to get familiar with interpreting a satellite image, ensuring that participants were comfortable with the nadir view. We identified urban features in the imag,e like residential building,s and differentiated between paved and unpaved roads. In the next step, participants used their smartphone to visit a web application^30^ that randomly pairs the sampled image tiles for participants to select which is the better place to live. There was no limit to the number of pairs a participant would vote on. However, the voting session was set to be done for one hour to allow the participants to get familiar with the application and gain some momentum in making their pairwise comparisons as they see more pairs. The voting session ended after an hour, regardless of the number of votes per participant.

The participants in the community workshops were recruited with assistance from the local community leaders, with gender and age diversity being the main considerations for participant selection. For the city planners’ workshop, we prioritized inviting people from diverse expertise backgrounds, including local government, academia, NGOs, and civic societies (all with expertise in urban planning).

### Training an AI-voter to simulate human choices

Using the pairwise comparisons gathered by the local participants, we trained deep learning models to simulate how to select the “better place to live” given two images. While the local participants were shown high-resolution Google Earth imagery during the workshops, our AI-voter was trained on the S1 images clipped to the sampled tiles of 20 pixels.

**Fig. 10:**
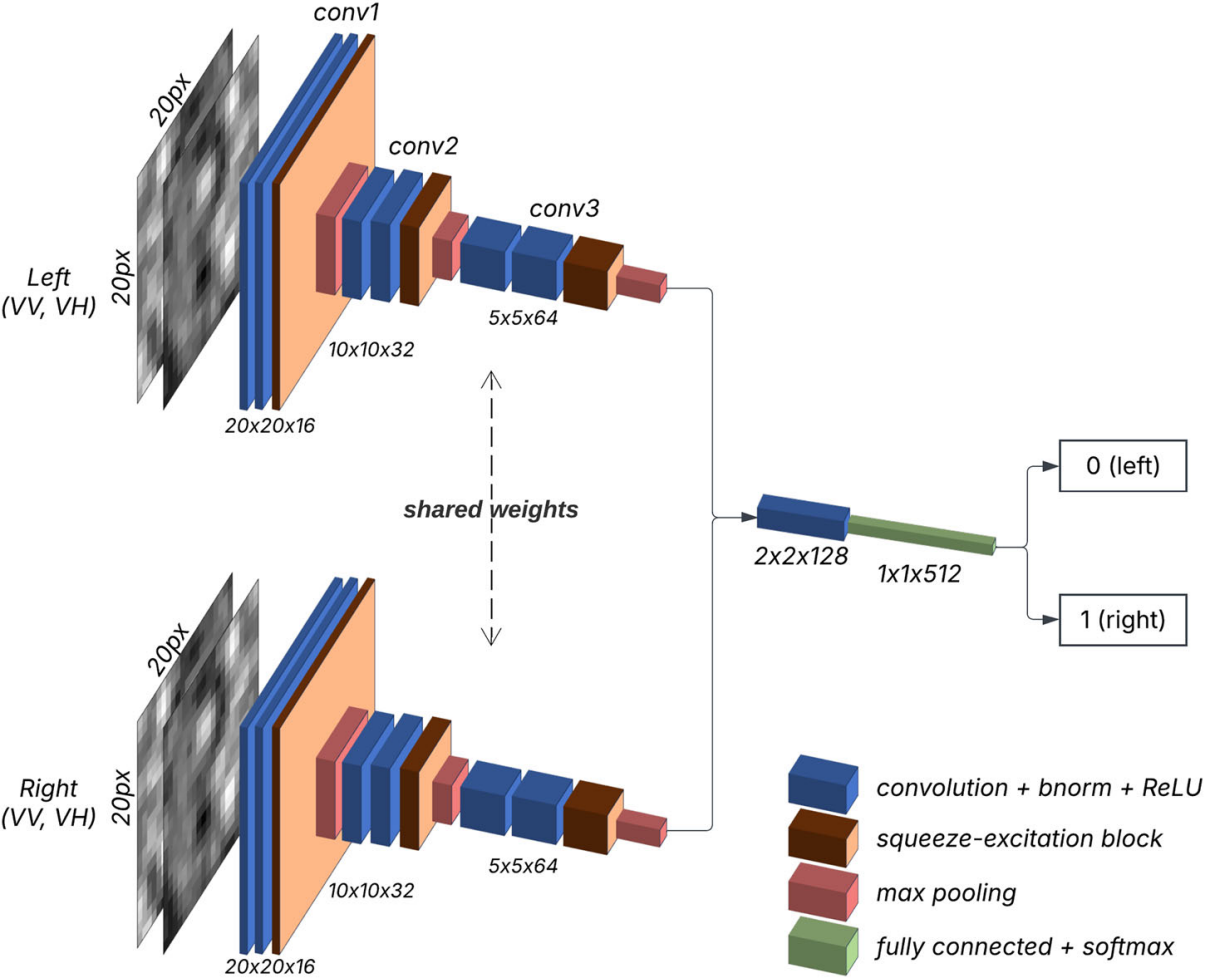
VoterNet – a modified and light Siamese Twin network architecture used as the AI-voter.

The network architecture of our AI-voter is a reframing of the Siamese Twin network, which was initially used for similarity detection^65^. The typical Siamese Twin network takes two input images and if the inputs are dissimilar, would output 0, and 1, otherwise. Our pairwise network (VoterNet), as seen in Fig. 10, has two modifications from a typical Siamese Twin network: 1) the two feature

extractors do not start with pretrained weights since we do not have a baseline task to train such weights, but are nevertheless trained together and shared, and 2) the interpretation of the label value (and predicted value) is that 0 means the left image was selected as the better place to live while 1 means the right image was selected. Given the tile size of 200 m and the spatial resolution of S1 imagery (10 m), VoterNet only had access to a limited number of pixels per input image (20px x 20px). Therefore, we built a shallow version of the Visual Geometry Group (VGG) architecture with only nine layers (VGG9) paired with squeeze-and-excitation (SE) blocks at each layer as a feature extractor. The shallow architecture eases the learning process for the model since fewer weights need to be learned from the limited number of pixels per image. A pair of S1 images would be passed to the AI-voter as input (left and right). These would go into a shared VGG9 feature extractor to learn low- and mid-level features from the inputs. The features were then fused and passed on to a single 3×3 2D convolution layer with 128 features before the final prediction was made via a softmax output layer. The output values were probabilities for both class 0 and 1. The class with the higher probability would be the final predicted class, while the probability of the final prediction was used as a confidence metric for that specific prediction.

We trained three AI-voters from scratch using VoterNet, varying only the sources of training data: votes from 1) the DUA residents only, 2) the city planners only, and 3) a combination of DUA residents and city planners. For the combination, since there were substantially more votes from the DUA residents than the city planners, we randomly sampled votes from the DUA residents equal to the total number of city planner votes to ensure equal influence from both sources. The sampled votes from the DUA residents were combined with the planners’ votes. All models also started with the same randomly initialized weights to ensure that only the training data was different. A 5-fold cross validation (CV) was performed so our test sets were independent of the training sets to ensure that our assessments were not over-optimistic. To assess the models, we calculated the binary accuracy for each fold. The selection of the loss function (Huber loss) and hyperparameters like learning rate (0.001) and optimizer (Adam) was done based on binary accuracy evaluation and learning from previous experiments. The 5-fold CV strategy resulted in five different trained AI-voters for each of the three sources of training data. To combine them and produce a single output prediction per training set, we trained an ensemble model that took the average of the predictions from each of the five CV models.

### City-wide livability maps from different perspectives

As the image pairs the humans selected from were only representative samples, to produce city-wide livability maps, we generated synthetic image pairs for all 6800 image tiles across the city and made the trained AI-voters vote on such pairs. To convert these pairwise comparisons into livability scores or rankings, we used TrueSkill^32^(TS), a Bayesian rating system that was originally made for online game rating but has also been used in studies on perception ranking^30,39,66^. In TS, the votes are treated as matches in a tournament where all players (image tiles) gain or lose rating as they win or lose matches, like in chess or online games. All image tiles start with the same rating μ, and getting selected as the better place to live constitutes a win and otherwise, a loss. At the end of the tournament, or when all votes have been made, TS provides an uncertainty σ along with the rating μ. The lower the σ, the more stable the rating. For reference, σ < 2 meant a relatively stable rating, while σ < 1 suggests a very stable rating^32^. Our initial experimentation showed that at least 98% of tiles reached very stable ratings (σ < 1) when each image was compared at least 200 times. This resulted in a total of approximately 860,000 unique synthetic image pairs for each AI-voter to vote on. Since the sequence of how the votes are ingested by TS affects the final ratings, we also ensured that all three AI-voters voted for the exact same set of pairs with the same order. For easier interpretation, we standardized the resulting ratings from 0 to 1, which represented the livability score of that tile for that specific AI-voter. We applied this procedure to all three trained models, resulting in a livability map based on the perception of 1) DUA residents, 2) city planners, and 3) their combination. The differences in raw livability scores between the DUA residents and city planners were calculated by subtracting the city planners’ livability scores from the DUA community's livability scores.

To identify spatial patterns within the livability scores, specifically clusters of low livability and high livability, we conducted a local Moran’s I hotspot analysis. We defined the spatial relationships of the tiles with a Queen contiguity spatial weights matrix, or neighboring regions as those that touch each other either along an edge or at a corner (8-directions), and identified clusters of low and high livability based on the spatial distribution of the livability scores at a significance level of 0.95. This was done for the three livability maps produced in the previous step. We compared the clusters from the DUA residents and city planners to see where their livability clusters matched and where they disagreed.

### Explaining locals’ and AI voting behavior and livability scores

We investigated whether any physical features had impacts on the locals’ and AI-voter’s choices. First, we let each AI-voter vote on the image pairs their human counterparts voted on. We then quantified the voting behavior of the different user groups and their corresponding AI-models by calculating the winning ratio of each image tile. This was done by counting the times an image tile was selected as the better place to live relative to the number of times it was paired to another tile. The relationship of this ‘winning ratio’ with physical features like green spaces and building density was analyzed as previous work has shown that these features relate to socioeconomic status^67,68^, which is related to PUL. To quantify such physical features for each tile, we computed the maximum NDVI, TCA, mean IBD. The maximum NDVI was derived from Sentinel-2 images of the same time, while the mean IBD was calculated from Google Open Buildings v3^62^. TCA was derived from the global canopy height dataset^69^ by filtering only the pixels with a canopy height above 1 m and calculating the total number of those pixels per tile. We calculated the correlation of each physical feature and the winning ratio for the three AI-voters as well as the human votes per user group.

## Data Availability

The datasets generated and/or analyzed during the current study are available in the GitHub repository (https://github.com/enzocampomanesv/ai-voter).

## Code availability

The underlying code for this study is available in the GitHub repository and can be accessed via this link (https://github.com/enzocampomanesv/ai-voter).
